# Research through Evaluation for Large Language Model in Patient-Clinician Communications

**DOI:** 10.21203/rs.3.rs-9735115/v1

**Published:** 2026-06-18

**Authors:** Yuexing Hao, Jason Holmes, Jared Hobson, Alexandra Bennett, Elizabeth L. McKone, Daniel K. Ebner, David M. Routman, Satomi Shiraishi, Samir H. Patel, Nathan Y. Yu, Chris L. Hallemeier, Brooke E. Ball, Saleh Kalantari, Marzyeh Ghassemi, Mark Waddle, Wei Liu

**Affiliations:** Massachusetts Institute of Technology; Mayo Clinic; Mayo Clinic; Mayo Clinic; Mayo Clinic; Mayo Clinic; Mayo Clinic; Mayo Clinic; Mayo Clinic; Mayo Clinic; Mayo Clinic; Mayo Clinic; Cornell University; Massachusetts Institute of Technology; Mayo Clinic; Mayo Clinic

## Abstract

Large Language Models (LLMs) demonstrate strong capabilities in healthcare applications. However, recruiting human evaluators to assess usability and effectiveness becomes challenging in the large-scale LLM-generated outcomes. Existing metrics are often outdated for evaluating LLM-based healthcare technologies. This paper proposes a Research through Evaluation (RtE) approach that can refine metrics and improve assessments of LLM-generated outputs. We deploy RtE in a retrospective comparative study of prostate cancer patient inquiries, comparing responses from human clinical care teams with those generated by a GPT-4-based in-basket bot. Through three rounds of co-evaluations with clinical professionals and an LLM-as-graders study with four LLMs, the RtE method shows that starting with small, iterative evaluations helps human graders adjust metrics to suit their daily practices better. Additionally, in the LLM-as-graders study, LLMs exhibit similar behavior, preferring the in-basket bot’s responses over the clinical care team. Our methodology and findings demonstrate that the RtE method can enhance the transparency and robustness of evaluation metrics for LLM-generated content, with the potential to generalize to evaluations in other clinical practices.

## Introduction

In Human-AI interactions, there are multiple conventional ways to evaluate a technical application^1–3^. However, despite the wide array of existing validated evaluation metrics and methods, researchers studying the emerging domain of LLMs have found it difficult to adapt the current evaluation methods to this new field^4,5^. Existing evaluation methods can sometimes overlook new problems arising from emerging technology, or lead to new features not being measured or measured properly^6,7^. Key aspects such as fairness, bias, toxicity, robustness, and deployment considerations have been largely neglected^8–11^.

Human-AI interaction typically relies on human evaluators, and the quantity and quality of the evaluation results often vary depending on the nature of each domain^12–14^. The inherent characteristics of a domain influence the challenges related to recruitment, training, user experience, sample size, and final prototype outcomes^15,16^. Subjective evaluations in this area are often affected by a lack of standardization, low reproducibility^17^, poor study design, and weak application of experimental and statistical methods^18–20^. Some domains are understudied, making it difficult to identify target populations due to privacy concerns or restrictions, and in some cases, recruited participants may not be appropriate evaluators for the developed prototypes. Thus, while the evaluation results are of significant importance, the development of robust evaluation processes is often overlooked.

The healthcare domain often involves missing or unstructured data, making the subjective evaluation of research outcomes challenging^21–23^. When tackling healthcare-related tasks, models require careful consideration of specialized evaluation metrics addressing causality, data gaps, and precise outcome definitions^24^. For clinical deployment, challenges like reproducibility and generalizability pose significant risks to apply or adapt an existing metrics, particularly due to the confidential nature of healthcare data and code accessibility^25^. Healthcare technologies are developed through multiple iterations, requiring rounds of development and evaluation to ensure alignment with the technology’s goals and to incorporate comprehensive, feasible measurements^26,27^. In a scoping review of usability testing on eHealth applications, Maramba and colleagues found that 41 out of 131 (31.3%) studies reported at least one further iteration of the app following usability testing results^28^. The zero-shot or one-shot evaluation is not applicable and not ideal in the LLM tool development in healthcare^29^.

Therefore, in the current paper we propose Research through Evaluation (RtE), a method to tackle the challenges of conducting subjective evaluations in healthcare. Starting with a new LLM or LLM-based prototype that has clear design goals and focusing on why the prototype is being developed, researchers can perform iterative evaluations to identify who the key stakeholders are, where biases exist, and refine evaluation metrics through ongoing exploration and experimentation^30,31^. While evaluations are often constrained by time and budget, our study focuses on conducting the RtE process by involving different types of stakeholders and leveraging multiple rounds of evaluation to build up rigorous evaluation metrics and processes.

The paper is motivated by three main research questions: (1) How can we adapt current evaluation metrics to better suit LLM applications in healthcare? (2) What role should human evaluators play in the RtE process? (3) What are the feasibility and perceived usability of the RtE process from the perspective of clinical professionals, and to what extent can the RtE process be automated with LLMs?

In this study, we involve a diverse group of evaluators in co-designing the evaluation process, with the aim of rethinking and updating evaluation methods to better align with the needs of emerging LLM applications in healthcare. The objective of this study focuses on assisting human evaluators in enhancing the quality of evaluations, including raising awareness of new problems as well as benefits that might emerge with these technologies, through a collaborative co-development approach.

## Results

### 1. Understanding Evaluation Settings in RtE Process

**Figure 1:**
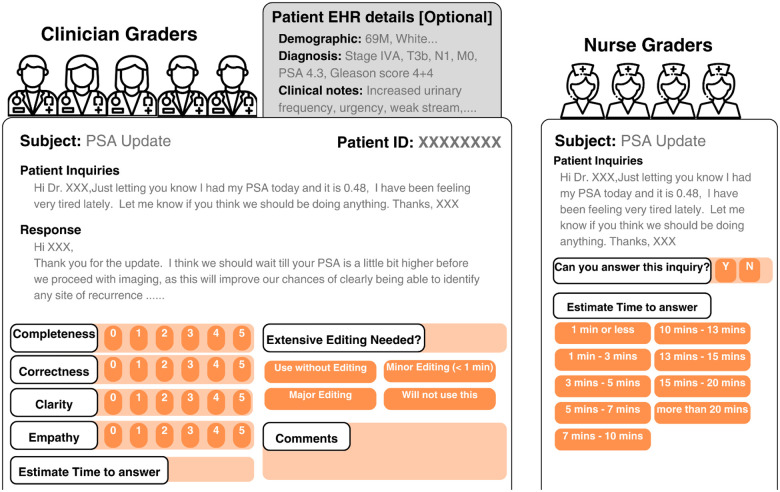
Overview of the grading process, involving clinicians and LLM graders. An example of the patient inquiry, the corresponding clinical team and in-basket bot responses are presented in the Supplementary Information.

We developed a GPT-4-based prototype, referred to here as the in-basket bot, which is already integrated with Mayo Clinic Electronic Medical Record (EMR) system, Epic, and the radiation oncology-specific Oncology Information System, Aria ver. 16. The RtE study validates whether this in-basket bot can generate responses that potentially enhance clinical efficiency and workflow. The in-basket bot has access to EMR databases and libraries, utilizing information such as family and medical history, lab results, diagnosis details, treatment plans, and clinical notes. Detailed information about the data accessible to the in-basket bot and its prompt is displayed in Supplementary Information. The full grading pipeline is in Fig. 1 and rubric is in Supplementary Information.

We retrieve data from the BigQuery Database, including all of the prostate cancer patients’ in-basket messages for the calendar years 2022–2024. The topics of these messages include questions about radiation treatments (i.e. proton therapy, photon therapy), screening services, or active disease surveillance, among other concerns. For our RtE study, we first select 90 specific nonmetastatic prostate cancer patients who had submitted questions to the care team from the dataset. We consider only messages that the patients had submitted under the “*Patient Medical Advice Request*”, as this category places less emphasis on logistical problems. We then filter out any inquiries that were not relevant to medical advice-seeking or did not receive a substantial reply from the human care team. This leaves us with 157 patient inquiries, along with the clinical care team’s replies to each message. We then submit each of these 157 patient inquiries to the in-basket bot and collected its responses. Due to the retrospective nature of the study and the presence of patient health information (PHI), patient participants were not involved. The in-basket bot was reinitialized before processing each query to eliminate memory effects from previous inquiries.

**Figure 2:**
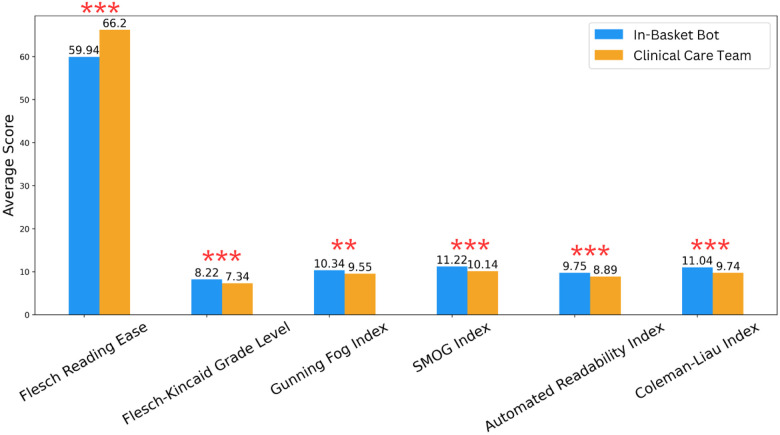
Comparisons of Readability Scores across Six Scales. In-basket bot-generated responses and Clinical Care Team responses exhibit systematic differences, with clinical team responses showing higher readability across all six scales. Stars indicate statistical significance (** p < 0.01, *** p < 0.001).

Our readability analysis compared the in-basket bot and clinical care team responses across six standardized scales, as shown in Fig. 2. On the Flesch Reading Ease scale, where higher scores indicate better readability, clinical care team responses score more favorably (66.2) than in-basket bot responses (59.9). For the remaining five indices: Flesch-Kincaid Grade Level^32^, Gunning Fog Index^33^, Simple Measure of Gobbledygook (SMOG) Index^34^, Automated Readability Index, and Coleman-Liau Index^35^, lower scores represent better readability.

Clinical care team responses consistently score lower across these metrics, indicating they’re written at more accessible reading levels. Structural analysis reveals characteristic differences in communication patterns. Though similar in overall word count (in-basket bot: 135, clinical team: 132), the clinical team achieved greater efficiency with fewer sentences per response (7 versus 9). The clinical care team’s responses are generally more readable. In contrast, the In-basket bot generates more complex responses across multiple readability indices. Since the dataset contains patient health information and we cannot recruit patients as evaluators, the readability analysis shows that the in-basket bot’s responses may require simplification to enhance accessibility, particularly for patients with lower health literacy.

To ensure robust grading results, our RtE study involves 4 rounds (Table 1): we first recruit two primary clinician graders (C1 and C2) evaluating a subset of the dataset to define an effective study design and estimate grading workloads. In the second round, we select evaluation metrics and develop grading rubrics. Building on existing literature and nurse practitioners’ recommendations, we focused on four key metrics: Completeness, Correctness, Conciseness, and Trustworthiness^36^. After the first two rounds of gradings, the researchers shared the results with all five clinician graders and conducted a round-table discussion regarding the metrics and the time required to evaluate.

In regard to the preliminary evaluations, C1 and C2 exhibited some differences in their assessments, with C2 generally showing a slightly more favorable evaluation towards the in-basket bot’s responses, especially in terms of Conciseness and Completeness. However, C1 tends to rate the bot’s responses higher for Trustworthiness. This highlights a subtle divergence in the evaluation criteria and the interpretation of the responses.

Additionally, C4 highlights that the in-basket bot’s primary goal is to alleviate the clinicians’ workload by automating tasks such as reading patient inquiries, reviewing the patient’s EHR or lab/exam results, and drafting responses. To address this, we introduced a new category, “*Extensive Editing Required?”*, to evaluate how extensive editing it should become.

### 3. Direct Comparison Study Results

In Round 1, disclosing both response types and comparing them simultaneously may have introduced bias. Clinician graders find it difficult to compare the two responses, even though they addressed the same patient inquiry: “*Neither the AI nor the human response was necessarily wrong; they just had different focal points*” (C3). To mitigate this bias, the clinician graders recommend a single-blinded randomized setup, where the source of the response would not be disclosed during grading. Although this would increase their workload, the evaluation set-up would help reduce bias, as direct comparisons between responses are challenging. *“The AI’s response was rarely incorrect, but comparing it directly to human responses was challenging*” (C1). C2 also suggests removing the “*Neutral*” option, as it is rarely selected, but other graders insist that “*Neutral*” could be valuable, especially when a senior grader needs to be involved.

After establishing a clear project goal, the grading criteria are initially unspecified, leading to disparities in how the two clinician graders interpreted the evaluation metrics. This variability is reflected in the inter-rater reliability analysis, where Correctness (ICC = 0.50) and Trustworthiness (ICC = 0.53) exhibit weak agreement, while Conciseness (ICC = 0.59) and Completeness (ICC = 0.64) show moderate but inconsistent alignment. Krippendorff’s Alpha values indicate variability (scores ranging from 0.30 to 0.61) suggesting differences in how the graders apply each metric. The detailed statistics are shown in Supplementary Table 1 and Supplementary Fig. 1. One grader (C2) highlights this challenge: “*If the AI response is wrong, even 1/3 wrong, it’s hard for graders to give it any positive marks*.” Meanwhile, C1 comments on a specific case: “*Overall, the human response is better, and I agree with everything up until the recommendation for Ibuprofen, which I would not recommend in someone with new/concerning urinary symptoms*.” These observations demonstrate the need for a structured rubric. The clinician graders collaborately develop a standardized grading rubric that generalizes across patient inquiries while clarifying ambiguous evaluation criteria.

### 4. Single-Blind Study Results

After identifying some bias in the first round of evaluation, we conduct another round of evlauation on 15 different pairs of patient questions and answers, allowing the two primary clinicians to implement the new metrics. Clinicians evaluate another small cohort of 15 pairs of responses, consisting of 15 bot responses and 15 clinical care team responses, all corresponding to the same 15 patient inquiries. The statistical results are presented in Supplementary Table 2, with visualizations provided in Supplementary Fig. 2.

Our inter-rater reliability analysis reveals varying degrees of agreement between the two graders across multiple dimensions. Correctness (ICC = 0.69) shows substantial agreement, while other dimensions exhibit poor to moderate alignment. The Krippendorff’s Alpha values reinforce these findings, with Correctness achieving higher agreement, while Completeness and Empathy reflect substantial inconsistency. These results suggest that rubric refinement is necessary to enhance evaluation consistency. The detailed statistical analysis are presented in Supplementary Table 2 and Supplementary Fig. 2.

After second round evaluation, we show the results to the five clinical evaluators and conduct another roundtable discussion on these results. One issue that emerges during second round of evaluation shows that clinicians encounter challenges when applying our grading rubric to specific cases. Clinicians particularly struggle with determining whether Correctness should strictly measure factual accuracy or account for broader clinical considerations. The observed variability highlights the need for additional training or structured calibration exercises in future iterations to improve inter-rater reliability. One of the clinicians noted an example of this: “*While AZO [a medication name for phenazopyridine] isn’t necessarily incorrect, I would generally recommend Flomax in my daily practice*” (C2).

Similar challenges emerged when assessing Completeness. We find that clinical care team responses are often less complete because they prioritize addressing the most urgent or high-priority questions, or direct patients to call for further discussion, which was not reflected in the dataset. In contrast, in-basket bot responses lacked sufficient depth, potentially due to the limited information provided in patient inquiries. The in-basket bot’s limitations stem from its reliance on the clinic’s database and patient inquiries, without the context of personal interactions that clinical care team members might have, such as a deeper understanding of the patient’s characteristics or medical history. As one clinician remarked in response to the in-basket bot’s advice on an itchy rash, “*No evaluation of the type of rash or any other symptoms to be concerned about. Just generic anti-itching advice. Also likely something the PCP should evaluate*” (C2).

Additionally, there are instances where the evaluation metrics overlap. A response could be considered complete by thoroughly addressing the patient’s inquiry but still provide incorrect medication recommendations or next steps. Conversely, a response might be incomplete but entirely accurate in the information it provided. To address these nuances, clinician graders develop a flexible grading rubric, which they could refer to during evaluations and expand with additional items as new or rare cases emerged.

Clinician graders also raise concerns regarding the category Trustworthiness. They express difficulty in assessing the trustworthiness of the responses from a patient’s perspective. In light of this feedback, the graders debate between replacing Trustworthiness with Empathy or Positivity. As one clinician noted, “*Empathy relates more to understanding the patient’s situation, whereas a positive response focuses on how many positive words are used*” (C1).

Clinician evaluators engage in discussions about the relationships between evaluation categories and the potential for bias from ordering effects. Overlaps between these categories could introduce such effects, where assessing one category first might influence the evaluation of another. To address this, the grading rubric is designed to ensure each evaluation item is distinct and has minimal overlap with other items. For instance, they debate whether a strong agreement for Completeness should necessarily imply a strong agreement for Correctness. It is possible for a response to be grammatically complete and address all of the patient’s questions without missing any elements, but still be incorrect for the specific patient’s case. Both clinician evaluators employ a strategy of reading the patient inquiry and response in full before assigning any ratings. The evaluation approach helps them to pull specific details or double-check the patient’s EMR profiles as needed.

### 5. Multi-Clinician Evaluation Study Results

**Figure 3:**
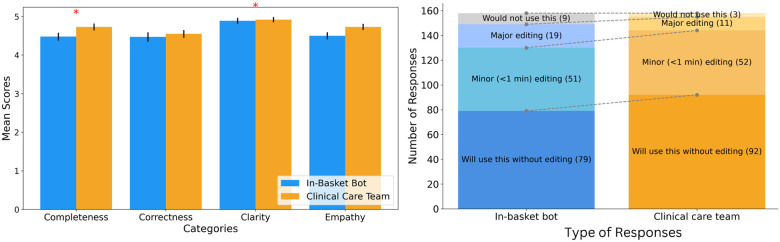
Detailed Results for Clinicians’ Final Grading. The left figure compares the final scores given by three clinician graders for the In-Basket Bot and the Human Care Team. The right figure outlines the time-saving strategies used by the In-Basket Bot compared to the Clinical Care Team. Sample outputs highlighting substantial performance differences between the Clinical Care Team and In-Basket Bot responses are available in Supplementary Table 3. (Note: * p < 0.05)

We then evaluate the feasibility and usability of the RtE process by evaluating 314 responses, 157 generated by the clinical care team and 157 by the bot in the box, using updated grading rubrics developed after Round 2. Three independent clinician graders (C1–C3) conducted the evaluation. Results indicate that the Clinical Care Team outperform the In-Basket Bot in Completeness, Correctness, and Clarity, while the In-Basket Bot demonstrate better performance in Empathy. Regarding time-saving strategies, the In-Basket Bot demonstrate fewer “*Will use this without editing*” responses compared to the Clinical Care Team but exhibite higher proportions of “*Major Editing*” and “*Would not use this*” responses. These results reflect a divergence in perceived usability and editing requirements. Messages generate by the In-Basket Bot often require more extensive editing before being suitable for sending to patients. The visualization of the detailed comparison is presented in Fig. 3.

To address discrepancies in grading, a fourth clinician (C4) reviewed 41 responses with significant variations among the primary graders, and a fifth clinician (C5) reviewed an additional two responses requiring further resolution. These secondary reviews were conducted in a randomized, single-blinded setup to ensure objectivity. Both C4 and C5 were familiar with the study’s rubric updates and prior grading iterations. Detailed profiles of the five clinician graders are provided in Table 2.

Clinicians are also surprised by the analysis results. In Round 1, the clinicians lean towards the responses of the clinical care team, but by the final classifications, the differences are minimal. “*I was confused about what and how to grade at first, but after adjusting the metrics and scales, it became much clearer*” (C2). Conducting small-scale Round 1 rather than grading everything at once made the evaluation process more efficient and less wasteful: “*Starting with a small cohort to train and validate was very helpful. I wouldn’t want to regrade everything after finishing it”* (C1).

Through first two rounds of gradings, two clinician graders have developed a clearer understanding of the responses, though the grading remains subjective. The clinicians appreciate the iterative discussions and the adaptation of metrics to align with their daily practice and the specific context of prostate cancer care. How should we position the subjective gradings? C2 remarks, “There is no objective ground truth. The in-basket bot’s response just brings it closer to how humans behave, for better or worse” (C2). The quality of the evaluation also improves as the grading rounds acted as a form of training. With more human evaluators involved in discussions, confusion decreases, and more considerations are made within the evaluation context. “*One round of grading isn’t enough to ensure fair and accurate evaluations. I suspect the initial confusion stemmed from trying to assess multiple criteria simultaneously*” (C3).

Clinician graders appreciate being involved in iterative evaluations and discussions around the evaluation criteria, definitions, and goals. Particularly in healthcare, where validation in clinical settings is the ultimate goal, clinicians value their participation: “*Involving us [clinician evaluators] in the evaluation process helps us understand whether something is worth evaluating and how to integrate it into our daily practice*” (C3). Determining the right role for evaluating specific questions is crucial. For example, the definition of Trustworthiness varies between patients and clinicians. “*It might seem appropriate from a patient’s perspective but not from a clinician’s point of view*” (C4). As a result, we replace Trustworthiness with Empathy, which emphasizes the compassionate tone clinicians convey to patients.

The goal of using the in-basket bot is to reduce clinician burnout from responding to patient inquiries. After reviewing the results, C1 notes, “*In-basket bot did well on empathy, which shows exactly why it is a great tool — provider burnout can lead to short, sometimes curt replies to patients. The in-basket bot allows providers to have a really nice starting base response (sometimes even the entire response without editing), and that is instrumental in combating provider burnout*” (C1).

### 6. LLM-as-A-Judge Validation Study Results

To evaluate whether different LLMs could match the evaluation quality of clinician graders, we use the same study setting as for the clinicians: randomized single-blinded grading, the same grading rubrics, and independent grading without knowing the clinicians’ scores. We test GPT-4, GPT-4o, Gemini 1.5-Pro, and Llama 3.1 on four criteria: Completeness, Correctness, Clarity, and Empathy. Other categories, such as “Estimate Time to Answer” and “Extensive Editing Needed?”, are reduced due to their subjectivity and the potential for random guessing without sufficient data training.

The LLMs are integrated with the EHR and provided with the same grading rubrics as prompts used by the clinician graders. We still use GPT-4 (the backbone engine of in-basket bot) to provide the gradings serving as a benchmark. We also test other LLMs from Gemini and Llama other than OpenAI to avoid data leakage or potential bias in the evaluation results.

**Figure 4:**
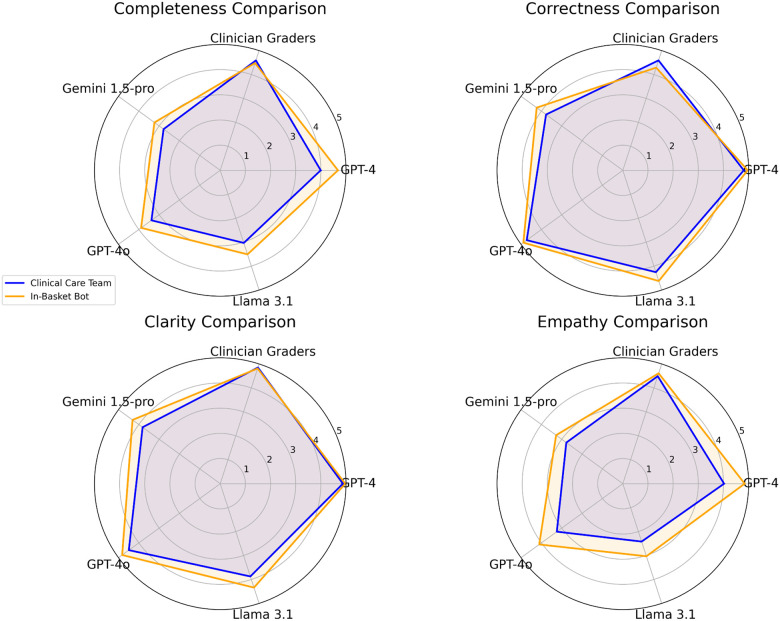
Radar chart comparing four dimensions across four LLM graders and the clinician average baseline. Using a zero-shot prompt engineering approach, GPT-4o rates in-basket bot responses with an average score of 17.70, and clinical care team responses with an average score of 15.83, both out of 20. Similarly, Gemini rates in-basket bot responses at an average of 15.05, and clinical care team responses at an average of 13.14, also out of 20. GPT-4 rates in-basket bot responses at an average of 17.7, for clinical team responses the average is 19.45. Llama 3.1 rates in-basket bot responses at an average of 15.5, for clinical team responses the average is 13.57.

GPT-4 graders consistently assign highest scores to GPT-4-based in-basket bot responses compared to clinician-drafted responses. The results suggest self-recognition bias where the model favors content generated by its own architecture^37^. Similarly, GPT-4 rates in-basket bot responses second highest. This pattern extends across all LLM families tested as Gemini 1.5-Pro and Llama 3.1 also favored in-basket bot responses across all four evaluation categories. In contrast, clinician graders only rate in-basket bot responses higher in the Empathy category. The results are shown in Fig. 4.

These discrepancies between LLMs and clinician evaluations reveal that LLM graders appear to prefer LLM-generated content which follows structured linguistic patterns resembling their training data. Since LLMs are trained on vast amounts of structured and well-formulated text, they may interpret the polished and concise nature of LLM-generated responses as superior, even when clinical team’s responses may be equally or more clinically appropriate.

We also interpret the benefits of this RtE method through inter-rater reliability to understand how RtE improves raters’ understanding of evaluation item concepts (Supplementary Table 3). Here, inter-rater reliability was assessed using a two-way random-effects model Intraclass Correlation Coefficient (ICC). Comparing grader types, the average rater ICC3k for LLMs is higher than for human graders, indicating that LLMs have more consistent grading mechanisms after receiving the evaluation metrics.

This discrepancy appears significant when examining reliability metrics. Clinician graders demonstrate substantially higher inter-rater reliability (Krippendorff’s Alpha: Completeness − 0.63, Correctness − 0.81, Clarity − 0.75, Empathy − 0.53) compared to LLM graders (Alpha: Completeness − 0.18, Correctness − 0.10, Clarity − 0.07, Empathy − 0.17). The striking contrast between high ICC3k values for LLMs (0.67 – 0.82) and their extremely low Alpha coefficients suggests systematic bias rather than genuine agreement. This pattern alongside highly significant p-values across all LLM graders (< 0.001 for most categories) supports the concern that LLMs recognize and preferentially score LLM-generated content regardless of clinical accuracy. When evaluation models systematically favor LLM-generated content based on stylistic rather than substantive qualities, we risk significantly overestimating LLMs’ capacity for authentic patient-centered care.

While LLMs tend to rate in-basket bot-generated responses higher than those from the clinical care team, they still score significantly lower than human graders (Supplementary Table 4). Notably, there is considerable variability between clinician and LLM grading, suggesting that the grading rubric is either too complex or not fully understood by the LLMs. Since answering in-basket messages in radiation oncology is a highly specialized domain and often requires domain-specific knowledge, implementing a few-shot or specifically trained LLM may be necessary to automate and improve the grading process.

## Discussion

Our work builds upon prior fair and transparent evaluation research by proposing a structured framework RtE that addresses the unique challenges of conducting subjective usability studies in digital health^38–40^. Unlike traditional evaluation approaches, RtE offers an adaptive process where evaluators continually refine their understanding of evaluation goals and address biases that emerge throughout multiple evaluation rounds. This approach builds on reflective design and the critical need for adaptive evaluation methods in complex environments, as highlighted by prior work in collaborative healthcare settings. The RtE approach provides a structured method to incorporate diverse perspectives and foster deeper evaluator engagement, contributing to a more rigorous and contextually aware evaluation process.

The iterative refinement central to RtE introduces procedural adjustments that should be carefully documented to maintain methodological integrity. Each evaluation round in RtE generates its own set of results. This is a transparent process that can be reviewed and replicated by other researchers, which strengthens research accountability and supports the generalizability of findings across evaluations. In our particular study context, where formal hypothesis testing was not conducted, these considerations manifest through our commitment to transparent documentation of the evaluation process and criteria evolution across iterations.

As the demand for expert graders grows and becomes increasingly scarce, automating the evaluation process with LLM graders presents a promising solution for providing prompt feedback. In our study, we examine zero-shot LLM graders and find notable differences compared to clinician evaluations. Particularly in critical areas like Correctness, LLM graders face challenges, including 1) lack of context, 2) insufficient domain-specific knowledge, 3) difficulty handling essential meta-tasks, and 4) hallucination issues (ranked by frequency).

Deploying LLM graders to replace human experts poses risks, as these graders show significant discrepancies across all standardized evaluation categories. LLM-as-a-grader may favor incorrect LLM-generated responses, which could be dangerous if scaled without expert oversight or auditing. Our findings reveal that zero-shot LLM graders consistently struggle to accurately assess healthcare responses. If LLMs are employed for both content generation and evaluation, their inability to detect and address errors could perpetuate systemic issues. These graders are poorly calibrated and exhibit a clear bias toward their own outputs. Using LLMs as decision-makers in this capacity risks amplifying these shortcomings and could result in negative outcomes due to their inherent self-preferential tendencies.

RtD method is limited by implicit knowledge and the challenges in documenting the emerged knowledge, such as methods, theories, and insights^41^. Our RtE methodology address these limitations through structured evaluator involvement from early stages, establishing clear checkpoints during initial discussions, and testing with focused datasets to validate study design. RtE effectively defines evaluation objectives and provides researchers clarity on how their prototype or intervention can provide value. Early evaluation failures offer opportunities to measure evaluator metric adherence through standardized measures like Krippendorff’s alpha, enabling before-and-after comparisons. Additionally, RtE optimizes evaluator time efficiency through multiple small-scale studies with limited evaluator groups and targeted datasets. By including evaluators in metric development, they gain deeper understanding of the criteria while simultaneously receiving training in protocol adherence.

Our study intentionally employs zero-shot LLM graders with prompt engineering to reflect current clinical reality. According to physician social network Sermo’s survey reports^42^, 76% of clinicians used general-purpose LLMs in clinical decision-making, not domain-specific or fine-tuned LLMs. Our LLM-as-a-grader study examines a workflow where LLMs generate preliminary in-basket message responses and evaluations using final RtE metrics. The findings show that while LLM evaluation saves time and costs compared to clinician review, LLMs display bias favoring LLM-generated content. This create substantial risk when using automated evaluation without clinical oversight. Such bias may undermine LLM reliability claims and confirms the essential role of clinicians’ expertise in clinical evaluation.

Our RtE process begins with a case study and offered insights for the broader LLM evaluation community. We encourage future researchers to implement iterative evaluation protocols and involve evaluators early in design and development phases. Our findings also demonstrate concerning biases as general-purpose LLMs consistently favored LLM-generated responses over human-generated content. The integration of LLM-as-a-grader into healthcare workflows remain premature and require further iterative testing before implementation. To mitigate the LLM-as-a-Grader’s bias^43,44^, RtE offers an optimal solution by validating human graders alongside with LLM graders results. RtE enables alignment to ensure LLMs accurately interpret evaluation criteria and domain-specific knowledge.

The evaluation process with human annotators is often constrained by dataset scale, time, and budget. In our study, we aimed to address these limitations through multiple iterations of grading to reduce potential biases. RtE approach allowed for a longer evaluation period, with human evaluators receiving more in-depth testing and training. However, this approach may not be suitable for all studies, as most studies’ participants only engage in short-term evaluations which makes it impractical to implement RtE in the study workflow. Additionally, the RtE method also incurs higher costs, as it requires repeated discussions and grading, limiting the size of the evaluator cohort. Future work could explore involving domain-specific LLM evaluators or incorporating few-shot training for LLM graders to determine whether the poor performance stems from the format of LLM-generated content or other specific factors lead to the poor grading results.

The goal of our RtE study is to mitigate specific limitations in the evaluation process. The human graders based on a small cohort of data may lead to bias, which has the possibility to drive a false change in the evaluation metric. Therefore, having discussions with different evaluators and only conducting small changes on the evaluation metric may help to mitigate the errors. Through our study as a starting point, future researchers can explore the RtE method in their own evaluation study designs, providing a framework for a deeper and concrete understanding of who should evaluate, when, how, and what to assess.

## Methodology

### 1. Case Study Design

To investigate the RtE process, we conducted a case study in collaboration with a radiation oncology department at a U.S. healthcare clinic. We conduct the IRB-approved RtE study at the Mayo Clinic Radiation Oncology department from June to December 2024 (ID: 24–013630), following the STROBE reporting guideline. All methods were carried out in accordance with the Declaration of Mayo Clinic and relevant institutional and federal regulations governing human subjects research. The requirement for informed consent was waived by the Mayo Clinic Institutional Review Board due to the retrospective nature of the study.

**Figure 5:**
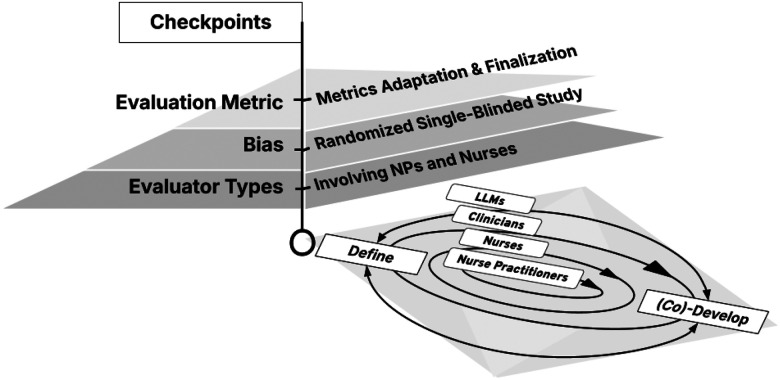
An overview of the RtE grading process involved clinicians, nurses, and LLM graders in the case study. The process follows a double diamond design structure (Structure C) in the X-Y plane, creating an iterative loop that alternates between defining questions and developing solutions. Multiple stakeholders, including nurse practitioners, nurses, clinicians, and LLMs (serving as graders or content generators), collaboratively develop both the questions and the evaluation metrics. In this study, the process starts from nurse practitioners to nurses, then to clinicians, and finally to LLMs, with each stakeholder participating in the define and (co-)develop phases of the double diamond. Along the Z-axis, multiple checkpoints are established throughout this iterative process to ensure the RtE grading methodology: (1) incorporates perspectives from all potential evaluators, (2) identifies and mitigates potential bias factors, and (3) refines evaluation metrics and verifies alignment with specific evaluation domains or fields.

We began with a fully developed LLM-based prototype, specifically an in-basket bot, with a focus on assessing how effectively the clinic’s chatbot could generate responses comparable to those of the clinical care team in addressing inquiries from prostate cancer patients. The clinic receives an estimated 5,000 patient inquiries per day, which is far more than can be reasonably handled by the clinic’s available resources, leading to staff burnout and delays in response times. The goal of implementing the in-basket bot was to expedite the response time while ensuring the responses remained accurate, complete, clear, and empathetic. To evaluate the in-basket bot’s performance, we employed the proposed RtE method to understand how human evaluators could meaningfully engage in the evaluation process and help finalize the evaluation metrics through the following three steps (as displayed in Fig. 5):

Step 1: Conduct qualitative research to understand initial challenges associated with various types of evaluators.

Step 2: Perform rounds of small-scale studies to refine evaluation metrics, addressing potential biases and making necessary adaptations.

Step 3: Conduct a final evaluation to assess the feasibility and usability of the developed approach.

Throughout these iterative evaluations and discussions, clinician graders gained a deeper understanding of the rubric, which helped to minimize potential biases during the RtE process. Unlike zero-shot or few-shot evaluations, incorporating continuous feedback through multiple iterations allowed both researchers and evaluators to better understand the evaluation objectives and address any areas that were previously misjudged or overlooked.

### 2. Evaluation Process Details

To ensure evaluators understand the evaluation metric and problems, we conducted iterations of evaluation round-table discussions.

During the second part of the interviews, we examined the primary challenges clinical professionals face when responding to patient inquiries and how an in-basket bot could enhance efficiency and accuracy. Time management emerged as a key concern: responses must be timely, yet providing quality medical advice involves validating patient information. For instance, “*prostate cancer patients probably need to wait 24–48 hours for a response*” (NP1), and delayed replies can intensify patient stress or lead to unnecessary follow-up visits.

Because these messages are considered medical advice, clinicians must devote extra effort to confirm the appropriateness of their recommendations. “*You have to evaluate the situation properly, ensure it’s an appropriate response, and approve it. Sometimes, it feels like you’re stuck in a black hole of in-basket responding*” (NP1). To mitigate these challenges, our proposed in-basket bot evaluation plan focuses on the speed and thoroughness of nurse responses, including time spent on reading, drafting, consulting the EHR, posing follow-up questions, or referring to clinicians. Meanwhile, the grading criteria for clinicians emphasize correctness (factual accuracy and patient relevance), completeness (responding to all inquiries), conciseness (personalized and specific communication), and trustworthiness (tone and context that bolster patient confidence). These metrics derive from the nature of medical texts and the characteristics of target end-user populations, aligning with the “Quality of Information” and “Trust and Confidence” dimensions outlined by Tam and colleagues. An optional comment section further enables graders to clarify any low scores or provide additional feedback.

We then consolidated the qualitative feedback into five key considerations in Table 2.

After reviewing the variability and conflicts in the clinicians’ assessments, we bring in two senior clinicians to perform the final evaluations. During the grading process, clinician graders have the option to review the patient’s EMR profiles based on the patient ID to ensure accuracy.

Clarity, Empathy, and Extensive Editing are evaluated holistically, with all key points consider together rather than assess independently. Clinicians are not required to assign separate scores to each key point. Instead, they provide a single score for each criterion based on their overall judgment of how well the response aligned with the guiding aspects outlined in the key points. This approach ensure a comprehensive evaluation without reducing the assessment to a mechanical checklist.

For Clarity, clinicians assess the response’s overall comprehensibility, logical structure, and accessibility to a layperson. They consider whether the language is easy to understand, whether medical terms are appropriately explained, and whether the response is well-organized. Similarly, for Empathy, they evaluate the extent to which the response acknowledges the patient’s concerns, conveys a compassionate and supportive tone, and makes the patient feel heard and cared for. The Extensive Editing criterion follows a structured 4-point scale, where clinicians determine whether the response could be used without modification, required minor or major edits, or was unsuitable for patient communication.

Since the key points within each criterion address differently, they serve as guiding principles rather than separate components requiring individual scores. Clinicians synthesize these considerations into a single representative score for each criterion, rather than averaging independent assessments of each key point. This approach allows for a clinician-driven evaluation that reflected both the content and tone of the responses.

NP1 and NP2 are nurse practitioner evaluators specializing in prostate cancer and urology, respectively. C1, C2, and C3 are primary clinician graders, while C4 and C5 are senior clinician graders. All NPs and clinicians are recruited from the clinic’s prostate cancer specialty to ensure consistency in the evaluation process. Each NP and clinician grader is compensated $70 for their participation in the grading study.

In our study, the three primary checkpoints are: evaluator type (after consulting with NPs, we finalized the evaluators as registered nurses, clinicians, and LLM-as-graders); bias reduction (from Round 1 to 2, we adjusted the clinician graders’ study design from direct comparison with disclosed sources to a single-blinded randomized approach); and evaluation metrics (from Round 2 to final clinician gradings, we replace 1) Conciseness with Clarity, 2) Trustworthiness with Empathy, and 3) added timing metrics to align with prototype development goals following discussions with stakeholders). These evaluation metrics, initially determined by clinicians, require iterative refinement to ensure they accurately reflect the study’s objectives. Table 3 details each evaluation study’s refinements regarding evaluator type, data quantity, study design methods, and specific metrics or annotation scales.

While RtE offers a systematic approach to evaluation, its iterative nature requires careful methodological considerations. This sampling approach differs from traditional AI or machine learning benchmarks that often establish fixed evaluation protocols on comprehensive datasets. RtE provides a foundation for rigorous assessment adaptable to other specialized domains requiring expert evaluation. The iterative nature of RtE allows for continuous refinement of evaluation criteria, keeping pace with evolving LLM capabilities.

### 3. RtE Approach

While most research studies position evaluation as the final stage to demonstrate improvements over existing datasets or interventions, they rarely explain how these evaluations align with the specific scope of their investigation. RtE’s goal is to help researchers involve diverse stakeholders to objectively look at the intervention or product in the early stage, document each step of the evaluation and outcomes to be external knowledge. Evaluating with a small cohort of evaluators and a subset of data can help to grasp an overview of third-party evaluators’ perspectives and avoid missing the important points early on.

**Figure 6:**
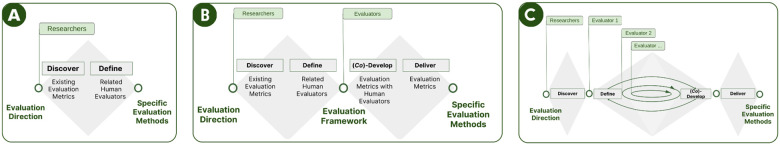
Three structures of iterative RtE processes from research direction to specific study methods.

We first start from a single-diamond framework (Fig. 6, Structure A). This Discover–Define structure is commonly used in current research: researchers discover existing evaluation metrics and define them with the help of recruited human evaluators, typically engaging evaluators at later stages. Then we add a new diamond to form a Double-Diamond framework (Fig. 6, Structure B), which comprises two more stages (Co)-Develop-Deliver to include two phases: the first question diamond, where researchers define the study direction, and the second solution diamond, where evaluators help refine study methods before the final evaluation methods are delivered.

Triple-Diamond framework (Fig. 6, Structure C) divides the process into three distinct diamonds: discover study direction, iterative framework exploration, and delivering final methodology, allowing earlier evaluator involvement and multiple types of evaluator engagement in the iterative process. RtE utilizes Structure C’s design, with the goal of exploring how researchers and human evaluators can jointly enhance evaluation quality at each stage of the research process.

Here, we propose the RtE grading process, defining as integrating evaluation early in the study design by incorporating checkpoints through iterative exploratory evaluations. This approach starts with preliminary evaluations using limited cohort datasets and a subset of evaluators. These initial evaluations serve to validate both the evaluation framework and the evaluator’s experience. During this iterative process, evaluation limitations, potential biases, and methodological incongruities emerge. The challenges facilitate discourse between researchers and evaluators on the type of evaluation, the evaluation design parameters (e.g., independent assessment, double-blind protocols, randomization strategies) and the selection of metrics. Following a synthesis of preliminary findings, the optimized evaluation protocol is implemented across the entire data set. The researchers subsequently analyze the final evaluation results and identify how the insights derived from the preliminary evaluations mitigated the possible methodological pitfalls. This structured approach constitutes the complete RtE process.

The RtE process balances evaluation freedom and prototype development with systematic validation in clinical environments. Implementing the RtE method in LLM’s study design can potentially make the evaluation process more rigorous, improve grading quality, and facilitate better adaptation to the field. This iterative approach in evaluation development allows each iteration’s findings to foster methodological understanding and interpretation of outcomes.

## Supplementary Material

This is a list of supplementary files associated with this preprint. Click to download.

- RtESupplementaryInformation.pdf

## Figures and Tables

**Table 1:** Overview of the RtE Process.

# Round & Evaluators	Pairs	Study Method Design	Metrics
Round 1 Grading - Graders C1, C2	30 Pairs	Direct Comparison: GPT vs. Clinician	Clinician Metrics: 1) More Completeness; 2) More Correctness; 3) More Conciseness; 4) Trustworthiness; 5) Other Comments
Round 2 Grading - Graders C1, C2	15 Pairs	Randomized Single-Blinded Study	Clinician Metrics (Scale 1–5): 1) Completeness; 2) Correctness; 3) Conciseness; 4) Trustworthiness; 5) Extensive Editing Required; 6) Other Comments
Round 3 Grading - Graders C1, C2, C3 - Grader C4 - Grader C5	158 Pairs (316 Responses) & 41 Responses & 2 Responses	Clinician single-blind evaluation of responses from in-basket bot vs. clinical care team	Clinician Metrics (Scale 1–5): 1) Completeness; 2) Correctness; 3) Clarity; 4) Empathy; Metrics (Scale 0–3): 5) Editing Level; Short Answer (Minutes): 6) Estimate Time to Answer; 7) Other Comments
Round 4 Grading - Graders GPT-4, GPT-4o, Gemini 1.5-pro, Llama 3.1	158 Pairs (316 Responses)	LLM automated evaluation to validate its grading capability in comparison with clinician graders	LLM Graders Metrics (Scale 1–5): 1) Completeness; 2) Correctness; 3) Clarity; 4) Empathy

**Table 2:** Design Considerations and Explanations

Design Consideration	Explanations
**Acknowledge the patient’s concern**.	At both the start and end of each response, it is crucial to “*recognize the patient’s worry or reason for messaging”* (NP2). Although healthcare providers routinely manage urgent issues, patients often lack similar knowledge or experience. Explicitly confirming their concerns can help them feel reassured.
**Structure complex inquiries**.	When multiple questions appear in a single message, NPs may “*break down responses into smaller sections*” (NP1). This step-by-step approach prevents overwhelming patients and ensures clarity.
**Standardize repetitive responses**.	To enhance efficiency, NPs employ smart phrases within the electronic health record (EHR) interface for common scenarios (e.g., dysuria, rectal bleeding, or sleep advice). While “*smart phrases provide clear next steps*” (NP2), slight modifications are often necessary to address individual circumstances.
**Verify patient details**.	Before finalizing a response, NPs “*check additional EHR information, such as family history, recent medications, and lab results*” (NP1). This verification remains vital, even when leveraging large language models (LLMs).
**Build trust**.	“*Clear, empathetic responses*” (NP1) and follow-up inquiries demonstrate that providers are “invested in the patient’s care” (NP2). Nevertheless, NPs must set boundaries by staying within their scope of practice to avoid complications.

**Table 3:** Evaluator Information: Nurse Practitioners (NP) and Clinicians.

Evaluator	Gender	Specialty	Average Responses Per Day / Experience with responding in-basket messages	Role
NP1	Male	Prostate Cancer	12–15 responses per day / 4 yrs	Nurse Practitioner
NP2	Male	Urology	18–25 responses per day / 12 yrs	Nurse Practitioner
C1	Male	Radiation Oncology	3–5 yrs	Primary Clinician Grader
C2	Female	Radiation Oncology	3–5 yrs	Primary Clinician Grader
C3	Female	Radiation Oncology	3–5 yrs	Primary Clinician Grader
C4	Male	Prostate Cancer	5–10 yrs	Senior Clinician Grader
C5	Male	Prostate Cancer	10–30 yrs	Senior Clinician Grader

## Data Availability

We release 157 pairs of anonymized patients’ chat histories with in-basket bot in Supplementary Data 1 and also can be download via https://yuexinghao.github.io/Research_Through_Evaluation/data.html. Also, a sample in-basket bot chat history graded by clinicians and LLM graders are provided in the Supplementary Table 5.

## Code Availability

Codes used for data processing and analysis were provided in the Supplementary Information. The full set of prompts, including retrieval functions, is also available in our public GitHub repository at: https://yuexinghao.github.io/Research_Through_Evaluation/code.html.
